# Baicalin protects the myocardium from reperfusion-induced damage in isolated rat hearts via the antioxidant and paracrine effect

**DOI:** 10.3892/etm.2013.1369

**Published:** 2013-10-29

**Authors:** FENG KONG, YUN LUAN, ZHAO-HUA ZHANG, GUANG-HUI CHENG, TONG-GANG QI, CHAO SUN

**Affiliations:** 1 Central Research Laboratory, The Second Hospital of Shandong University, Jinan, Shandong 250033, P.R. China; 2 Department of Pediatrics, The Second Hospital of Shandong University, Jinan, Shandong 250033, P.R. China

**Keywords:** baicalin, ischemia-reperfusion, FCM, apoptosis, heart

## Abstract

The aim of the present study was to investigate the protective effect of baicalin (BA) against ischemia-reperfusion (I/R) injury in isolated rat hearts. Sprague-Dawley rat hearts were rapidly removed, mounted on a Langendorff apparatus and subjected to 30 min ischemia followed by 30 min reperfusion with Krebs-Henseleit (K-H) solution at 37°C to establish the isolated I/R injury model. All animals (n=50) were randomly divided into five groups (n=10 in each): I, normal control; II, I/R; III*,* I/R plus 20 mg/kg BA; IV, I/R plus 40 mg/kg BA; and V, I/R plus 80 mg/kg BA. The degree of heart injury caused by the I/R was assessed by evaluating left ventricular function and by detecting the levels of lactate dehydrogenase (LDH) and creatine kinase (CK) in the coronary effluent and the myocardial superoxide dismutase (SOD) and malondialdehyde (MDA) levels in the isolated rat hearts. Myocardial infarct size and vascular density were assessed using histology and immunohistochemistry. The apoptotic cardiomyocytes were determined using flow cytometry (FCM). Compared with group II, the BA groups demonstrated improved left ventricular function, reduced CK and LDH release in the coronary effluent and increased SOD and MDA activity (P<0.05). Furthermore, histology and immunohistochemistry results showed that the infarct size was reduced and vessel density was augmented in the BA groups (P<0.01) compared with group II. The FCM results indicated that apoptosis was significantly lower in the BA groups than in group II (P<0.05) and that the protective effect was dose-dependent. In conclusion, these results demonstrated that BA exerts a dose-dependent protective effect on I/R injury in isolated rat hearts, the mechanisms of which may be associated with antioxidant and anti-apoptosis properties. To the best of our knowledge, this study is the first evaluation of the efficacy of BA in isolated rat hearts using histology and immunohistochemistry, providing a foundation for the use of BA in the treatment of acute myocardial infarction.

## Introduction

Ischemia-reperfusion (I/R) injury remains the leading cause of morbidity and mortality in all developed countries and is a large economic burden in the treatment and care of patients (1). I/R injury of the heart is the underlying pathophysiology of acute myocardial infarction (AMI), which is one of the most common causes of mortality globally (2). Following cell I/R, apoptosis is one of the major pathways that leads to the process of cell death. Numerous studies have indicated that cell apoptosis is associated with a variety of damaging stimuli, particularly continuous I/R, *in vitro* and in the intact heart *in vivo*(3–7). Thus, protection against I/R injury in the heart is of marked importance.

Chinese herbal medicine has been used to treat diseases for thousands of years and it has recently attracted the attention of practitioners of Western medicine. However, the effective ingredients in the majority of these medications have not been identified. Baicalin (BA; C_21_H_18_O_11_, 7-glucuronic acid, 5,6-dihydroxy-flacone), a Chinese traditional medicinal herb, possesses antioxidant properties and free radical scavenging activity (8–12). Studies have demonstrated that BA can suppress the proliferation of vascular smooth muscle cells (9) and exert cardioprotective effects against hypoxia/reoxygenation injury (12–16). However, the cardioprotective properties have not been confirmed and the mechanism has not been fully characterized. In addition, there has been no clear histological or immunohistochemical evidence showing the cardioprotective effect of BA to date. Therefore, further studies investigating the mechanisms of protection are required.

The Langendorff mouse heart model is widely used in studies of myocardial function and responses to injury (17). In the present study, rat hearts were isolated and perfused using the Langendorff technique to establish a high-throughput and potentially reliable I/R model.

The aim of the present study was to evaluate the efficacy of BA in isolated rat hearts using hemodynamic, histological and immunohistochemical observations.

## Materials and methods

### Animals

Male Sprague-Dawley rats, weighing 250–300 g, were purchased from the Experimental Animal Center of Shandong University (Jinan, China). The rats were housed within the animal care facility at a constant room temperature with a 12:12-h light-dark cycle and were provided with standard rat chow and water *ad libitum* for at least 3 days prior to the initiation of the experiments. All animals received humane care in compliance with the Guide for the Care and Use of Laboratory Animals published by the US National Institute of Health (NIH publication no. 85-23; revised 1996). The study was approved by the Ethics Committee of the Shandong University (Jinan, China).

### Langendorff isolated perfused heart preparation

Rats were anesthetized with pentobarbital sodium (50 mg/kg ip) and administered with the anticoagulant, heparin sodium (10,000 USP U/kg ip). Following this, a thoracotomy was performed and the hearts of the rats were rapidly excised into ice-cold arresting solution (120 mmol/l NaCl and 30 mmol/l KCl). The aorta was cannulated on a 20-gauge stainless steel blunt needle and perfusion was initiated at 70 mmHg on a Langendorff apparatus. A modified Krebs-Henseleit (K-H) perfusion solution was used in all experiments, containing 118 mmol/l NaCl, 4.7 mmol/l KCl, 2.5 mmol/l CaCl_2_, 1.2 mmol/l MgSO_4_, 1.2 mmol/l KH_2_PO_4_, 24 mmol/l NaHCO_3_, 5.5 mmol/l glucose, 5.0 mmol/l sodium pyruvate and 0.5 mmol/l EDTA bubbled with 95% O_2_-5% CO_2_ at 37°C (pH 7.4). Electrodes were placed in the upper left and lower right regions of the heart and a left ventricular catheter with a pressure transducer (TP-400T; Nihon Kohden Corp., Tokyo, Japan) was connected to the Medlab Biosignal Acquisition System (MedLab-U/4C501, Top Instrument Co., Ltd., Hangzhou, China), to record heart rate (HR), left ventricular end-diastolic pressure (LVEDP), left ventricular developed pressure (LVDP), mean coronary flow (CF) and the first derivative of the left ventricular pressure during a cardiac cycle (maximum and minimum LV dP/dt). Heart pacing at 360 beats/min was initiated following 30 min stabilization to normalize HR across the groups. All hemodynamic parameters were continuously recorded on an eight-channel thermal-pen recorder (WT-685G; Nihon Kohden Corp.). Coronary effluent samples were obtained at 5, 15, 25, 35, 45 and 55 min of perfusion, and stored at −20°C for the measurement of lactate dehydrogenase (LDH) and creatine kinase (CK) activity. At the end of reperfusion, the left ventricular free wall was rapidly excised and stored at −80°C for subsequent determination of the levels of antioxidant enzymes.

### Experimental protocols

BA (purity >95%) was purchased from Sigma (St. Louis, MO, USA) and dissolved in saline prior to being added into the perfusion solution. All hearts were perfused with the K-H solution for a total of 120 min (at 37°C), consisting of a 30-min preischemic period followed by 30 min of ischemia and 60 min of reperfusion. The hearts were randomly divided into five experimental groups. Group I (normal): Hearts (n=10) were perfused for 90 min with K-H solution as a normal control for the different experimental groups. Group II (I/R): Following equilibration, hearts (n=10) were subjected to ischemia for 30 min, prior to being reperfused for 60 min with K-H solution. Groups III, IV and V (I/R plus BA): Hearts (n=10 each) were perfused similarly to group II, except that the reperfusion solution contained 20, 40 and 80 mg/kg BA, respectively.

### Assessment of myocardial damage

Activities of CK and LDH in the coronary effluent were measured by a 722 Visible Spectrophotometer (Shanghai Laipade Science Instruments Co., Ltd, Shanghai, China) using a Sigma assay kit at 340 nm, as described previously (18). Samples of the perfusate and the coronary effluent were collected following 5, 15, 25, 35 and 60 min of reperfusion and frozen in liquid nitrogen.

The malondialdehyde (MDA) level and superoxide dismutase (SOD) activity in the frozen myocardial tissue were measured using commercial kits (Nanjing Jiancheng Bioengineering Institute, Nanjing, China) with the 722 Visible Spectrophotometer at 532 nm (19). The cardiac tissue samples were weighed and homogenized (1:10, w/v) in 50 mmol/l phosphate buffer and maintained in an ice bath. The amount of thiobarbituric acid-reactive substances was estimated as MDA and SOD equivalents per gram of wet myocardial weight.

### Evaluation of myocardial infarct size

Following reperfusion and the rapid excision of the heart, the tissues were fixed with 10% formaldehyde, cut transversely, from apex to base, into six slices and embedded in paraffin. Serial sections (1 cm) of the embedded tissue were stained with hematoxylin-eosin and Masson’s trichrome stain (Baso Biotechnology, Shenzhen, China) and the amount of surviving myocardium was measured using Masson’s stain. For each slice, the area at risk and the area of infarction were determined by the sum of the planimetered endocardial and epicardial circumferences of the infarcted area divided by the sum of the total endocardial and epicardial circumferences of the left ventricle, as described previously (20,21).

### Analysis of vessel density

The hearts were rapidly harvested and the tissues in the infarcted zone were collected. The tissues were subsequently embedded in optimal cutting temperature compound (Sigma), rapidly frozen in liquid nitrogen and stored at −80°C. Having been fixed in acetone for 10 min at 4°C, cryostat sections were then cut into 5-μm slices and incubated with primary antibodies. Immunohistochemical staining was performed with an antibody against von Willebrand factor (vWF; 1:100; Abcam, Cambridge, UK), in accordance with the manufacturer’s instructions. The vascular density was counted blind on 100 sections in the infarcted areas of all animals and then stained with an anti-vWF antibody, using a light microscope at a magnification of ×400. The average of the 10 high-power fields (hpfs) was randomly selected and the vascular density was defined as the number of vessels/hpf in the infarcted area (0.2 mm^2^). Samples were randomized and two examiners, blind to treatment design, were used for analysis.

### Assay of apoptosis

Cardiomyocyte apoptosis was analyzed via flow cytometry (FCM) using a FACSCalibur system with CellQuest acquisition software (BD Pharmingen, Inc., San Diego, CA, USA) (22). Briefly, a single-cell suspension the from minced tissue of the LV free wall was obtained by mechanical grinding, prior to filtration through 40-μm nylon mesh filters (Falcon Cell Strainers; BD Biosciences Discovery Labware, Bedford, MA, USA). FCM analysis was performed using an Annexin V-fluorescein isothiocyanate (FITC) kit (Immunotech, Beckman Coulter, Miami, FL, USA) according to the manufacturer’s instructions: 500 μl 1X Binding Buffer was mixed with 10 μl propidium iodide (PI) and 10 μl Annexin-V labeled with FITC solutions. The cells were washed twice with ice-cold phosphate-buffered saline and then incubated for 15 min in 100 μl fresh incubation buffer containing PI and FITC-Annexin-V in the dark at room temperature. Following this, the cells (>1×10^6^/ml) were analyzed as soon as possible (within 1 h) using FCM.

### Statistical analysis

All data are presented as the mean ± standard error of the mean. A Student’s t-test was used to compare the data between two groups and one-way analysis of variance was used to compare the data from more than two groups, followed by the Scheffe multiple-comparison test. Statistical analyses were performed using SPSS version 13.0 statistical software (SPSS, Inc., Chicago, IL, USA). P<0.05 was considered to indicate a statistically significant difference.

## Results

### Effect of BA on hemodynamics and LV function

There were no significant differences in the baseline values of the cardiovascular parameters, including LVEDP, LVDP, CF and maximum and minimum LV dp/dt, between any of the groups. BA infusion caused virtually no changes in any of the measured cardiovascular parameters prior to ischemia in the ischemic groups. During the 30 min ischemia, LVDP, CF and maximum and minimum LV dp/dt decreased in all the groups. At 60 mins of reperfusion, these parameters showed partial recoveries in the I/R groups. Recoveries of LVDP, CF and maximum and minimum LV dp/dt were significantly improved in groups III, IV and IV compared with those in group II, in a dose-dependent manner (P<0.05). LVEDP was significantly increased during the 30 min of ischemia, and increased again at the end of the 60-min K-H solution reperfusion period (P<0.05). However, the increases in LVEDP during reperfusion in groups III, IV and V were significantly smaller than those in group II, as previously reported (23).

### Effect of BA on LDH and CK in coronary effluent

In all the groups, the activities of LDH and CK in the coronary effluent prior to ischemia were minimal. However, following ischemia, in group II, releases of LDH and CK into the coronary effluent were significantly increased compared with the normal group and significantly decreased in groups III, IV and V compared with group II (Table I).

### Effect of BA on SOD and MDA in myocardial tissue

The level of myocardial MDA was significantly higher and SOD was significantly lower in the ischemia groups than those in the normal group (P<0.01). However, the levels were significantly improved in groups III, IV and V than those in group II, with the results showing a dose-dependent effect (Table II).

### Infarct size and vessel density analysis

**Figure 1:**
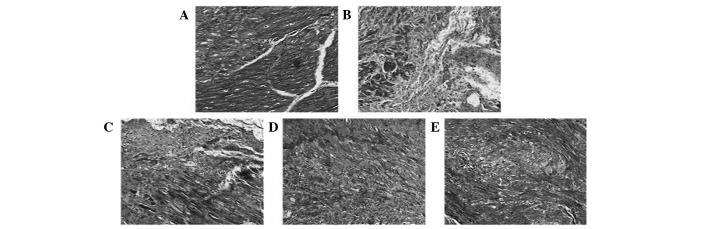
Effects of BA on myocardial infarct size visualized with Masson’s trichrome. Compared with the (A) control group, the infarct size was significantly increased in (B) group II. By contrast, it was significantly decreased in groups (C) III, (D) IV and (E) V compared with group II (magnification, ×100). Group I (control): Hearts were perfused for 90 min with K-H solution as a normal control for the different experimental groups. Group II (I/R): Subsequent to equilibration, hearts were subjected to ischemia for 30 min followed by reperfusion for 60 min with K-H solution. Groups III, IV and V (I/R + BA): Hearts were perfused similarly to group II, except that the reperfusion solution contained 20, 40 and 80 mg/kg BA, respectively. BA, baicalin; K-H, Krebs-Henseleit; I/R, ischemia-reperfusion.

**Figure 2:**
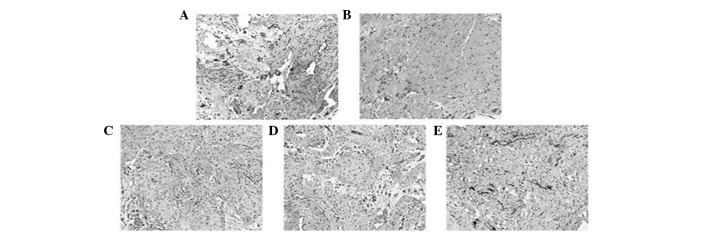
Effects of BA on vessel density. Vessels were stained with von Willebrand factor. Compared with the (A) control group, the density of the vessels was significantly reduced in (B) group II. By contrast, the density was significantly increased in groups (C) III, (D) IV and (E) V compared with group II (magnification, ×100). Group II (I/R): Subsequent to equilibration, hearts were subjected to ischemia for 30 min followed by reperfusion for 60 min with K-H solution. Groups III, IV and V (I/R + BA): Hearts were perfused similarly to group II, except that the reperfusion solution contained 20, 40 and 80 mg/kg BA, respectively. BA, baicalin; K-H, Krebs-Henseleit; I/R, ischemia-reperfusion.

The infarct size was significantly increased in group II (65.3±4.2%) compared with the control group (10.6±3.5; P<0.001). Furthermore, the infarct size was significantly decreased in groups III (43.8±2.7), IV (38.7±2.3) and V (35.4±3.1) compared with group II (P<0.05, Fig. 1). The vessel density was significantly reduced in group II (5.3±1.05) compared with the control group (12.7±1.08; P<0.001), while it was significantly increased in groups III (8.2±1.36), IV (9.6±1.24) and V (10.4±1.09) compared with group II (P<0.05, Fig. 2)

### Apoptosis detected by FCM

Following cell labeling with Annexin-V-FITC and PI, FCM analysis of the cardiomyocytes revealed that the percentage of apoptotic cells following I/R was significantly increased compared with normal cells (20.33 vs. 23%). Following BA treatment (20, 40 or 80 mg/kg), the percentage of apoptotic cardiomyocytes was significantly increased (P<0.05) in a dose-dependent manner (18.32, 14.95 and 10.12%, respectively).

## Discussion

*Scutellaria baicalensis* Georgi is a widely used herb in traditional medical systems of China and Japan (24). Flavonoids, including baicalein, BA, wogonin and skullcap flavones I and II, are major components of *Scutellaria baicalensis* Georgi and have been suggested to exert antioxidant and other pharmacological effects. The variety of interesting effects exhibited by BA has led to it attracting considerable attention and it has been widely used in oriental medicine. Studies have previously demonstrated the protective effects exerted by BA in myocardial ischemia in the isolated rat heart and against hypoxia/reoxygenation damage in rat cardiomyocytes (12–16). The mechanisms behind this protection may be due to the antioxidant, prooxidant and anti-inflammatory effects of BA, induced by the hypoxia/reoxygenation injury to cardiomyocytes. However, the mechanisms are still not fully understood and, therefore, further studies into the mechanisms of protection are required.

I/R injury remains the leading cause of morbidity and mortality in all developed countries and is a large economic burden on the treatment and care of patients. Following cell I/R, apoptosis is one of the major pathways leading to cell death (25,26). Programmed cell death, in the form of apoptosis, necrosis and, possibly, autophagic cell death are the final arbiters of cardiomyocyte numbers following myocardial infarction (27–29). Apoptosis has been ascribed a pathogenic role in I/R, particularly in the heart and brain (30,31). It has been indicated that cell apoptosis is associated with a variety of damaging stimuli, particularly continuous I/R. Therefore, the inhibition of cardiomyocyte apoptosis induced by myocardial I/R injury in the heart is particularly important for reducing myocardial damage.

In the present study, an isolated Langendorff-perfused rat heart model was used to evaluate the protective effect of BA against I/R injury. The heart was exposed to ischemia for 30 min and then reperfused with K-H perfusion solution for 60 min to establish an I/R model *in vitro*. It has previously been shown that BA is able to reduce LDH leakage and increase the survival rate of cardiomyocytes undergoing I/R (7). In our experiment, the activities of LDH and CK in the coronary effluent in the I/R group were significantly increased compared with those in the normal group. Furthermore, LDH and CK activities were significantly decreased in the BA groups, compared with group II, in a dose-dependent manner (P<0.05). By contrast, the myocardial MDA and SOD levels in the BA groups were significantly lower than those in group II (P<0.05). Our results indicated that the protective effects of BA against I/R injury were mediated by its antioxidant activity.

As mentioned previously, studies have demonstrated that BA is able to suppress the proliferation of vascular smooth muscle cells and exert cardioprotective effects against hypoxia/reoxygenation injury. However, the mechanisms are complicated, and, to date, there has been no clear histological and immunohistochemical evidence showing the cardioprotective effects of BA. To further study the protective effect of BA, the efficacy of BA was evaluated in isolated rat hearts using histology and immunohistochemistry and cardiomyocyte apoptosis was measured using FCM. The results showed that infarct size was reduced and vessel density was augmented in the BA groups (P<0.01). Labeling the cardiomyocytes with Annexin-V-FITC and PI showed that the percentage of apoptotic cells in I/R injury was significantly increased compared with normal cells (P<0.05). Following BA treatment (20, 40 and 80 mg/kg), the percentage of apoptotic cardiomyocytes was significantly increased (P<0.05) in a dose-dependent manner.

In conclusion, our data suggest a dose-dependent protective effect of BA against I/R injury in isolated rat hearts and indicate that the mechanisms of the protective effect may be associated with antioxidant and anti-apoptotic properties. To the best of our knowledge, this study is the first evaluation of the efficacy of BA in isolated rat hearts using histology and immunohistochemistry and may provide a theoretical foundation for the clinical treatment of AMI. Further studies in this field are must be performed.

## Figures and Tables

**Table I:** Effect of baicalin on LDH and CK activity in the coronary effluent.

			Reperfusion, U/ml					
			
Group	Parameter	Preischemia	5 min	15 min	25 min	35 min	45 min	55 min
II	CK	33±2.6	58±3.7	60±4.5	62±1.7	63±6.3	65±4.5	68±5.8
	LDH	2.38±0.2	10.27±0.3	10.26±0.1	9.48±0.2	9.25±0.5	9.16±0.1	8.70±0.4
III	CK	32±3.7	57±5.1	55±4.9^a^	49±8.1^a^	42±3.4^a^	38±2.6^b^	36±1.4^b^
	LDH	2.43±0.6	9.61±0.7	8.13±0.4^a^	7.25±0.8^a^	6.38±0.9^b^	5.10±0.4^b^	4.90±0.6^b^
IV	CK	34±2.8	56±3.8	55±2.6^a^	47±6.3^a^	39±1.8^b^^c^	37±4.7^b^^c^	35±2.1^b^^c^
	LDH	2.36±1.7	9.37±1.3	7.37±1.3^a^	6.28±1.2^a^	5.34±1.2^b^^c^	4.60±0.8^b^^c^	3.60±0.7^b^^c^
V	CK	32±4.2	56±2.6	52±2.4^a^	45±1.7^a^	36±4.9^a^^c^	34±4.7^b^^c^^d^	33±4.5^b^^d^
	LDH	2.36±1.7	9.24±1.3	7.26±2.0^a^	5.80±3.2^a^	4.80±1.3^b^^c^^d^	4.20±0.8^b^^d^	3.40±1.7^b^^d^

Data are presented as the mean ± standard error of the mean (n=10).

a P<0.05 and

b P<0.01, vs. group II;

c P<0.05, vs. group III;

d P<0.05, vs. group IV.

LDH, lactate dehydrogenase; CK, creatine kinase.

**Table II:** Effect of baicalin on SOD activity and MDA content in I/R-induced myocardial tissue.

Parameter	Control	Group II	Group III	Group IV	Group V
SOD (U/g)	441.3±14.3	180.6±23.7^a^	246.7±19.5^b^	345.4±13.4^a^,^b^,^c^	408.0±18.6^a^,^b^,^c^,^d^
MDA (mmol/g)	61.2±5.8	270.7±3.8^a^	206.2±7.6^a^,^b^	148.4±5.1^a^,^b^,^c^	80.7±4.1^a^,^b^,^c^,^d^

Data are presented as the mean ± standard error of the mean (n=10).

a P<0.05, vs. control;

b P<0.05, vs. group II;

c P<0.05, vs. group III;

d P<0.05, vs. group IV.

SOD, superoxide dismutase; MDA, malondialdehyde; I/R, ischemia-reperfusion.
